# Potential antiprostatic performance of novel lanthanide-complexes based on 5-nitropicolinic acid

**DOI:** 10.1007/s00775-024-02054-0

**Published:** 2024-05-08

**Authors:** Amalia García-García, Pablo Cristobal-Cueto, Tania Hidalgo, Iñigo J. Vitórica-Yrezábal, Antonio Rodríguez-Diéguez, Patricia Horcajada, Sara Rojas

**Affiliations:** 1 Department of Inorganic Chemistry, Faculty of Science, University of Granada, Av. Fuente Nueva S/N, 18071 Granada, Spain; 2 Advanced Porous Material Unit, IMDEA Energy Institute, Av. Ramón de La Sagra 3, 28935 Móstoles, Madrid, Spain

**Keywords:** 5-Nitropicolinic acid, Lanthanide, Coordination compounds, Single-crystal X-ray diffraction, Prostate cancer

## Abstract

Two new lanthanide-complexes based on the 5-nitropicolinate ligand (5-npic) were obtained and fully characterized. Single-crystal X-ray diffraction revealed that these compounds are isostructural to a Dy-complex, previously published by us, based on dinuclear monomers link together with an extended hydrogen bond network, providing a final chemical formula of [Ln_2_(5-npic)_6_(H_2_O)_4_]·(H_2_O)_2_, where Ln = Dy **(1)**, Gd **(2)**, and Tb **(3)**. Preliminary photoluminescent studies exhibited a ligand-centered emission for all complexes. The potential antitumoral activity of these materials was assayed in a prostatic cancer cell line (PC-3; the 2nd most common male cancerous disease), showing a significant anticancer activity (50–60% at 500 μg·mL^−1^). In turn, a high biocompatibility by both, the complexes and their precursors in human immunological HL-60 cells, was evidenced. In view of the strongest toxic effect in the tumoral cell line provided by the free 5-npic ligand (~ 40–50%), the overall anticancer complex performance seems to be triggered by the presence of this molecule.

**Graphical Abstract:**

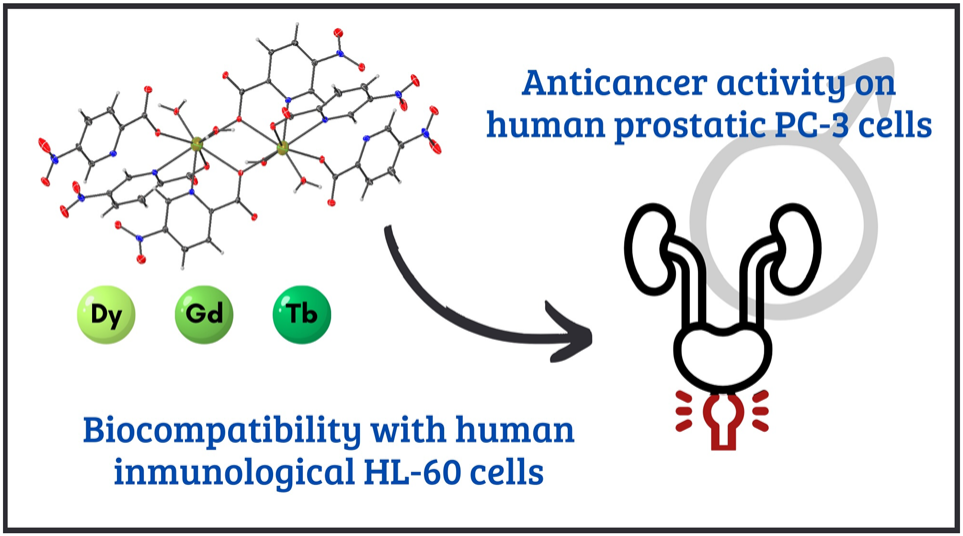

**Supplementary Information:**

The online version contains supplementary material available at 10.1007/s00775-024-02054-0.

## Introduction

Cancer is one of the leading causes of death worldwide, compromising more than 20 million people with nearly 10 million deaths worldwide only in 2020 [1, 2], remaining still as one of the biggest global health challenges in the twenty-first century. Particularly for men, prostate cancer is the second most frequent type of cancer, just after lung carcinoma, with ~ 1.3 million new diagnosis each year worldwide, making it a serious health concern [3]. Currently, early-stage prostate cancer treatments include prostate removal, hormone and/or radiotherapy [4, 5], which are associated with numerous side effects such as urinary or erectile dysfunctions [6]. In advanced-stages, the frequent tumoral metastasis and/or the appearance of resistance to classical chemotherapeutic agents lead to inefficient therapies or prognosis. The current used chemotherapeutic agents (e.g., mitoxantrone, estramustine, docetaxel) show short average lifetime with a great resistant generation rate [7]. In this sense, European Union (EU) has fostered a renewed commitment for cancer prevention, treatment and care for improvement the patient´s quality life through novel missions and actions based on repurposing medicines, underpinning data sharing or reinforce collaborations (e.g., Europe’s Beating Cancer Plan). One of these approaches in recent years has been the use of metal complexes in chemotherapy [8, 9], as for instance platinum complexes like oxaliplatin or cisplatin [10–12], some of the most successful anticancer compounds clinically used for diverse cancers (ovarian, gastrointestinal, bladder, etc.). For that reason, the relevance of metallodrugs as therapeutic and imaging diagnostics has exponentially risen in recent years: since cisplatin (1978) to numerous emerged coordinated compounds, such as carboplatin for treating ovarian carcinoma, ^99m^Tc-exametazime as an imaging agent in inflammatory bowel disease, or bismuth potassium citrate, currently evaluated in clinical trial for COVID-19 treatment. Particularly, trivalent lanthanide ions exhibit unique photoactive characteristics, including precise emission spectra for exceptional color purity, wide emission band that encompass the UV–Vis and near-infrared regions, a diverse range of lifetimes spanning from microseconds to seconds, remarkable luminescence quantum efficiencies which makes them suitable for combining diagnostics and therapy [13]. Further, they present a very low cytotoxicity, making them ideal for their application in biomedicine. Among them, few lanthanide-based compounds were able to undergo clinical trials, such as ^177^Lu-labeled anti-prostate-specific membrane antigen (PSMA) monoclonal antibody J591 in radiotherapy against metastatic castration-resistant prostate cancer (in phase II) [14], motexafin gadolinium for photodynamic therapy against brain metastasis (in phase III) or gadofosveset trisodium (based also on Gd^III^) for imageology of blood vessels (in phase IV) [15].

In our search for more efficient and potent metal-based compounds to combat diverse types of cancer [16–18], 5-nitropicolinic acid has been chosen for the design and preparation of novel coordination compounds. Picolinic acid is a metabolic by-product of L-tryptophan catabolism, exhibiting additional physiological effects such as anti-proliferative, immunological and neuroprotective features [19]. In particular, its anticancer properties have been already demonstrated through its ability to disrupt the cell proliferation, triggering the programmed cell-death or, even, being able to arrest the cell cycle in cancer cells [20]. Further, in an attempt to improve its antitumor activity, we decided to use the nitro-derivative, as nitro-group possesses a strong electron attracting ability that creates localized electron-deficient sites with molecules and interacts with biological nucleophiles present in living systems, such as amino acids [21]. Importantly, some nitro-group-containing drugs (i.e., misonidazole [22], pimonidazole [23], niclosamide [24]) present interesting antitumor properties, particularly in prostate cancer.

In view of this scenario, we have focused on the synthesis of new picolinic-derived complexes with potential antitumoral activity against prostate cancer. In an effort to create a flexible platform for imaging and treatment purposes, lanthanides with known luminescent and/or antitumoral effects have been selected as the metal coordination group [25, 26]: starting from the compound based on Dy^III^ [27], previously reported by our group and complemented by two new isostructural complexes based on Gd^III^ and Tb^III^ (i.e., [Ln_2_(5-npic)_6_(H_2_O)_4_]·(H_2_O)_2_; 5-npic = 5-nitropicolinate; Ln = Dy^III^ **(1)**, Gd^III^ **(2)**, Tb^III^ **(3)**). Their physicochemical characterization was performed using a set of experimental techniques (e.g., Fourier-transform infrared (FTIR), thermogravimetric analysis (TGA), fluorescence and UV–Vis spectroscopies and X-ray diffraction). Finally, their safety and antitumor activity were evaluated in vitro using human promyelocytic HL-60 and prostatic tumoral PC-3 cell lines, respectively.

## Experimental

### Materials and experimental techniques

All reagents were purchased from commercial sources (Sigma-Aldrich, Merck Group, Darmstadt, Germany) and were used as received without additional purification. Fourier transformed infrared (FTIR) spectra were measured in solid state on a Bruker Tensor 27 FT-IR in the range of 4000–400 cm^−1^, and Opus software was used as a data collection program. Thermogravimetric analysis (TGA) of solid samples were performed on a Shimadzu mod. TGA/50H analyzer. Samples were heated from 28 to 950 ºC at a heating rate of 10 ºC·min^−1^ under air atmosphere. Routine X-ray powder diffraction (XRPD) patterns were collected on a BRUKER D8 DISCOVER diffractometer equipped with a PILATUS3R 100 K-A detector and using Cu K*α* radiation (*λ* = 1.5406 Å). The XRPD patterns were registered with a 2*θ* range from 3° to 45° with a step size of 0.02° and scan rate of 30 s *per* step. Stability profiles in phosphate buffered saline (PBS) were measured by UV–Vis absorption on a Cary 100 UV–Vis spectrophotometer (Agilent Technologies, CA, USA) at a scan rate of 600 nm·min^−1^. Fluorescence spectra in solid state at room temperature were carried out in a Varian Cary-Eclipse spectrofluorometer (Agilent Technologies, CA, USA) at a scan rate of 120 nm·min^−1^.

### Synthesis of metal complexes

Compounds **1–3** were obtained by following a hydrothermal route. Firstly, 0.06 mmol of 5-npic ligand were dissolved in 2 mL of distilled water. In a separate vial, 0.02 mmol of the corresponding lanthanide salt (Dy(NO_3_)_3_·6H_2_O, Gd(NO_3_)_3_·6H_2_O, or TbCl_3_·6H_2_O) were dissolved in 1 mL of distilled water. Then, the lanthanide salt was mixed over ligand solution and the closed vial was introduced in the oven at 95 ºC. After 48 h, suitable crystals for single-crystal X-ray diffraction (SC-XRD) were obtained. The obtained crystals were filtered off in air atmosphere and washed with distilled water. The reproducibility of the synthesis was demonstrated by X-ray powder diffraction (XRPD; Fig. S1).

### Single-crystal X-ray diffraction refinement and crystallographic data

For all compounds, diffraction intensities were recorded on a Bruker APEX-II CCD with a photon detector equipped with graphite monochromated Mo K*α* radiation (*λ* = 0.71073 Å). The data reduction was carried out with APEX2 software [28] and corrected for absorption with SADABS-2016/2 [29]. The structures were solved by direct methods and refined by full-matrix least-squares with SHELXL-2018/3 [30] by using OLEX2 1.5 software [31]. The refinement parameters are listed in Table S1. Details of selected bond distances and angles are given in Tables S2–S3 (see Supporting information-SI). CCDC numbers are 2300374 and 2300375 for **2** and **3**, respectively.

### Stability assays

The chemical stability of the obtained complexes was determined in phosphate buffered saline (PBS) solution at 37 ºC by measuring the release of 5-npic ligand by UV–Vis spectroscopy. A 0.1 mM solution of each compound was prepared in 30 mL of PBS and incubated under bidimensional stirring at 37 ºC. At different incubation times (0, 0.25, 0.5, 1, 2, 3.5, and 5 h), an aliquot of 15 mL was extracted and the same volume of PBS was added to the suspension in a way to keep sink conditions. All kinetic studies were carried out in triplicate (*n* = 3; see SI-Sect. 4 for further details).

### Cell culture and cellular viability (MTT assay)

Human prostatic adenocarcinoma cell line PC-3 (ATCC^®^, CRL-1435™) was selected to evaluate the antitumoral capacity in comparison with a non-tumoral cell line, the human promyelocytic cells HL-60 (ATCC^®^, CCL-240™). Both cell lines were cultured in RPMI 1640 (Roswell Park Memorial Institute 1640) medium supplemented with 10% fetal bovine serum (FBS) and 1% penicillin/streptomycin (P/S) at 37 °C and 5% CO_2_ atmosphere. Cell lines were passaged twice a week at an 80% of cellular confluence (8 × 10^4^, ∼1 × 10^5^ cells *per* cm^2^), being harvested by trypsinization (1% trypsin–EDTA solution).

For the cytotoxicity assays, compounds **1–3** along with their metallic precursors and the organic ligand 5-npic were evaluated by an MTT (3-(4,5-dimethylthiazol-2-yl)-2,5-diphenyltetrazolium bromide) colorimetric assay. The cell line was seeded 24 h before in 96-well plates at a density of 1 × 10^4^ cells *per* we*ll* in RPMI medium supplemented with 10% FBS and 1% P/S. Treatment suspensions were prepared as dilution series with cell culture media (30 µL of the sample in aqueous solution were added to a final volume of 300 µL in RPMI media) achieving different decreasing concentrations diluting from 500 to 8 µg·mL^−1^. Then, treated cells were cultured for 24 h at 37 °C with a 5% CO_2_ atmosphere. Subsequently, the MTT reagent was added (5 mg·mL^−1^ in PBS) and incubated at 37 ºC for 2 h. Afterwards, MTT was removed, adding 100 µL of dimethyl sulfoxide (DMSO) to each well for 10 min. Finally, absorbance was determined at λ = 539 nm. The percentage of cell viability was calculated by the absorbance measurements of control growth and test growth in the presence of the formulations at various concentration levels.

## Results and discussion

### Structural description of metal complexes

**Fig. 1:**
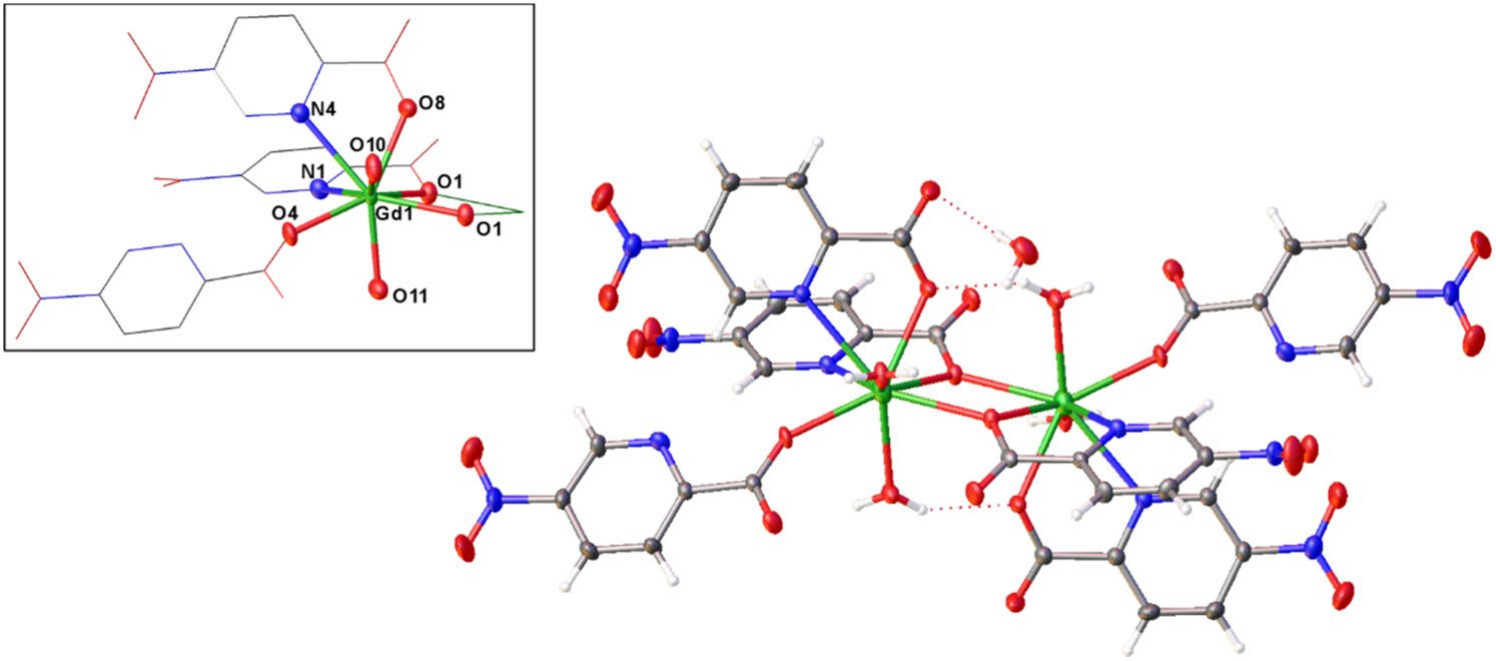
Isolated dinuclear entity [Gd_2_(5-npic)_6_(H_2_O)_4_]·(H_2_O)_2_ and its detailed coordination sphere. Color code: gadolinium, green; carbon, grey; nitrogen, blue; oxygen, red; and hydrogen, white

Compounds **1–3** are isostructural materials with the general formula [Ln_2_(5-npic)_6_(H_2_O)_4_]·(H_2_O)_2_, where Ln = Dy **(1)**, Gd **(2)**, and Tb **(3)**. Compound **1** has already been described by Raya-Barón et al*.* [27], thus, only compounds **2** and **3** will be here deeply described. The materials crystallize in the monoclinic space group *P*2_1_/*c*, which consist of Ln^III^ dinuclear complexes connected by an intricated hydrogen-bond network (Fig. 1).

The asymmetric unit is composed by one lanthanide ion, three coordinated 5-npic ligand molecules, two coordinated water molecules, and one crystallization water molecule. Ln^III^ center is coordinated to four oxygen atoms from four carboxylate groups of different ligand molecules, two water molecules, and two nitrogen atoms belonging to two different pyridine rings, creating a LnO_6_N_2_ coordination polyhedron. The Ln-O_carb_ bond distances are in the range of 2.296(4)–2.489(4) Å for **2** and 2.285(3)–2.471(3) Å for **3**, whereas the Ln-N_pyr_ distances are 2.558(5) and 2.655(5) Å for compound **2**, and 2.545(4) and 2.648(4) for **3** (Tables S2–S3), all in accordance with other similar complexes [32–34]. In these materials, the ligand exhibits three different coordination modes, thanks to which, Ln^III^ ions are connected by two μ_2_-oxygen atoms of two monodentated carboxylates ligands to form the dinuclear entity. The formed rhombus contains a crystallographic inversion center in the middle and impose an intra-dinuclear Ln–Ln distance of 4.0984(11) and 4.0805(8) Å for **2** and **3**, respectively.

**Fig. 2:**
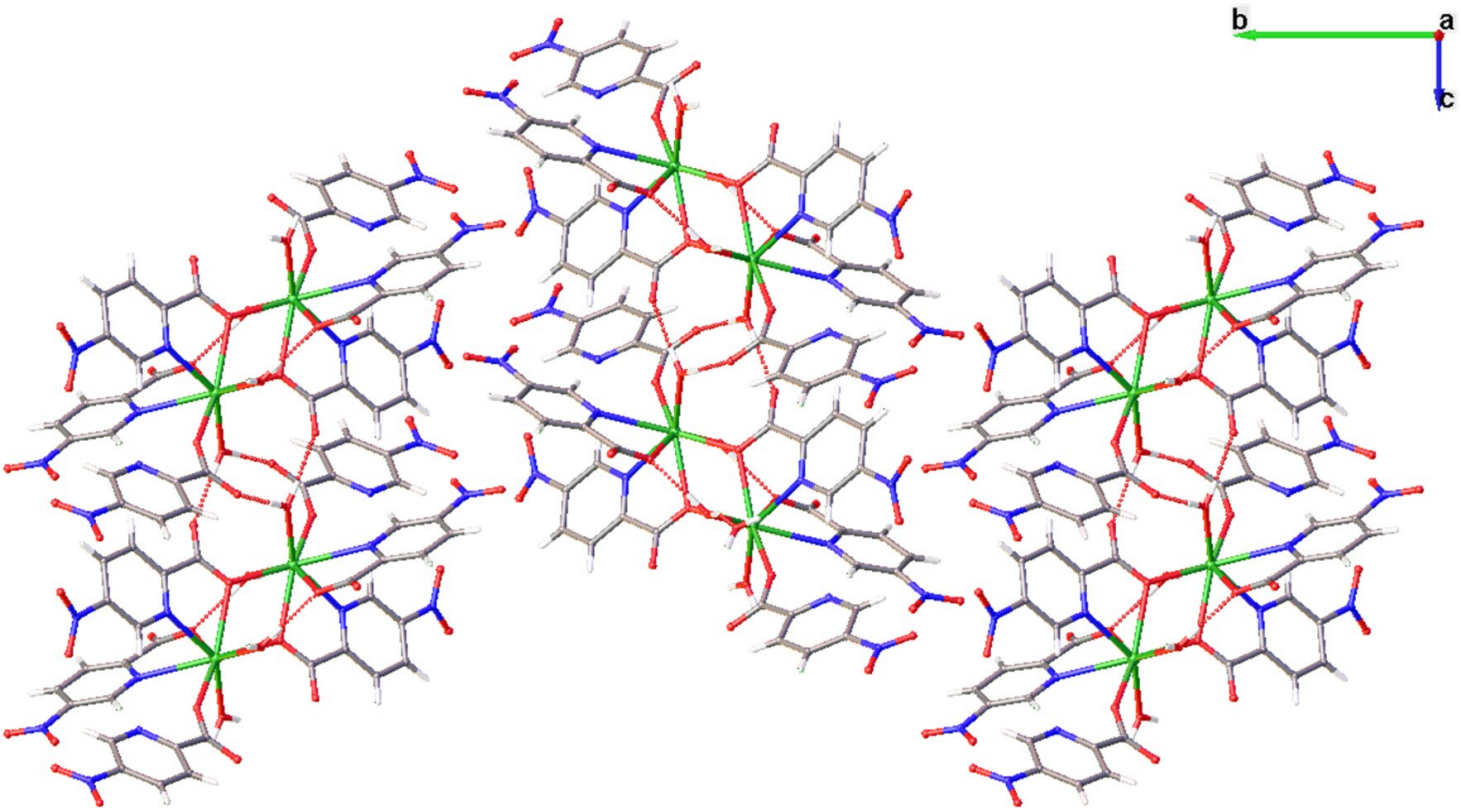
The dinuclear entities are packed one another along the *a* and *c* axis by an intricate hydrogen bond network (red dotted lines). In addition, glide planes parallel to *b* axis create a like zig-zag 3D assembly. Color code as Fig. 1

Within the structure, inter- and intramolecular interactions assist to stabilize the structure (Fig. 2). There are two intramolecular O···HO hydrogen bonds (2.759 and 2.754 Å for **2** and **3**, respectively) that involve one carboxylate group coordinated to one Ln^III^ atom and one water molecule coordinated to an adjacent Ln^III^ center. Neighboring dinuclear entities form a 1D chain network along the crystallographic *c* axis extended by O···HO hydrogen bonds with distances in the range of 2.64–2.677 and 2.647–2.669 Å for **2** and **3**, respectively, corresponding to the second coordinated water molecule and two oxygen atoms belonged to the adjacent carboxylate groups. Finally, these 1D chains are packed along *a* axis by O···HO hydrogen bonds involving the crystallization water molecules creating a 2D hydrogen bond network. In addition, glide planes perpendicular to *b* axis create a like zig-zag 3D assembly.

### Infrared spectroscopy

**Fig. 3:**
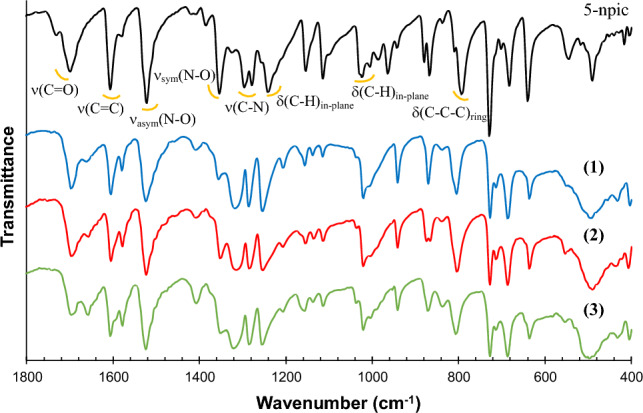
FTIR spectra of 5-npic and compounds **1–3** in solid state and at room temperature

The infrared spectra of complexes **1–3** and 5-npic ligand were registered in solid state at room temperature (Fig. 3). The characteristic bands of aromatic C–H bonds are observed in the ligand spectra at 3090–3078 cm^−1^, being nearly constant in the complexes’ spectra, as would be expected according to linker position in the crystalline structures. The peak correlated to stretch vibration of the C=O bond of the carboxylic acid is shifted from 1699 to 1695 cm^−1^ for 5-npic ligand and lanthanide complexes, respectively. This is indicative of the coordination between the lanthanide ions and the organic linker by this functional group. In addition, the peaks at 1518 and 1354 cm^−1^, attributed to the respective asymmetric and symmetric vibrations of the N–O bond, did not shown any variation, which suggests that the nitro group is not involved in any type of interaction with the ligand. The next intense band at 1296–1278 cm^−1^ is found in the ligand spectrum, which corresponds to the stretching vibration of the C–N bond of the pyridine ring, being shifted to 1317–1284 cm^−1^, since the nitrogen atom of the ring is also coordinated to the lanthanide ions.

### Luminescent features

Several coordination complexes based on diverse picolinic derivatives have already proven appealing photoluminescent properties on its picolinate skeleton [35–37]. Regarding the inorganic core, the potential application of lanthanide complexes in cancer therapy and diagnosis are growing great interest as a result of their luminescent and magnetic properties [38]. In view of these previous attributes, solid-state excitation and emission spectra of the novel compounds **1–3** and their 5-npic ligand were investigated at room temperature for polycrystalline samples.

**Fig. 4:**
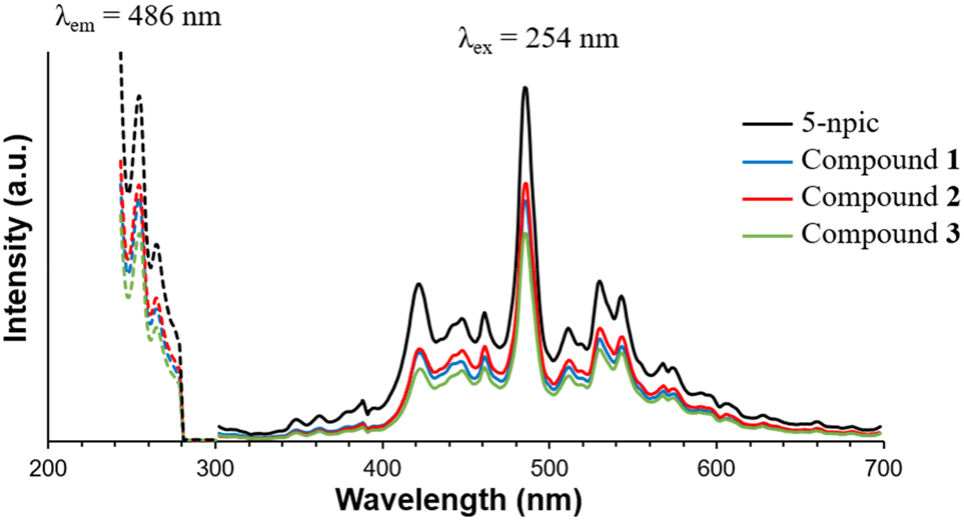
Excitation and emission spectra of compounds **1–3** and free 5-npic ligand (solid state) at room temperature

The linker emission is maintained for all complexes, with almost identical spectrum with a slight intensity (Fig. 4). The excitation spectra (recorded at λ_em_ = 486 nm) are characterized by two maxima peaks at 254 and 264 nm. Upon excitation at the maximum λ_ex_ = 254 nm, the emission spectra show several bands with an intense peak at 486 nm. Considering that complexes’ spectra were practically identical compared to the free ligand, the emission process of **1–3** could be attributed to a ligand-centered mechanism. These results demonstrate the potential of these as-prepared compounds as attractive fluorescence antitumoral molecules.

### Stability properties

Bearing this in mind, a key feature for their effective anti-proliferative or luminescent action is the investigation of the material stability prior to any in vitro assay. In other words, the potential leaching of their active constituents to the physiological environment («its degradation process») in a buffered solution. To this effect, the chemical stability of the prepared compounds was investigated by UV–Vis spectroscopy over the time. As depicted in Fig. S4, all lanthanide-complexes exhibited a similar degradation profile, with a fast degradation profile (100% of linker release in 5 h in PBS). At shorter times, the phosphate presence on the surroundings seems to affect in a similar manner to all the lanthanides complexes with a rapid initial release of the 5-npic (55 ± 9%, 40 ± 6% and 61 ± 6% in 30 min for **1**, **2** and **3**, respectively, namely as «*burst effect*»). The first 5-npic release data (0–1 h) were fitted to a zero-order kinetic (Fig. S4)., describing drug release at a constant rate independent from its concentration in the media [39, 40]. Based on the calculated rate of release constants (*K* = 0.0114, 0.0152 and 0.0103 h^−1^ for **1**, **2** and **3**, respectively), compounds **1** and **3** exhibited a similar behavior, whereas the linker released in **2** showed a slower leaching to the media. This could be explained by the increase in the atomic numbers and the corresponding decrease in ionic radii (Gd^III^ > Tb^III^ > Dy^III^), which significantly affects the bond strength [41]. Finally, and considering the Pourbaix diagram [42], the metallic species will be released as trivalent lanthanide ions to the solution considering the working conditions.

### In vitro biocompatibility

**Fig. 5:**
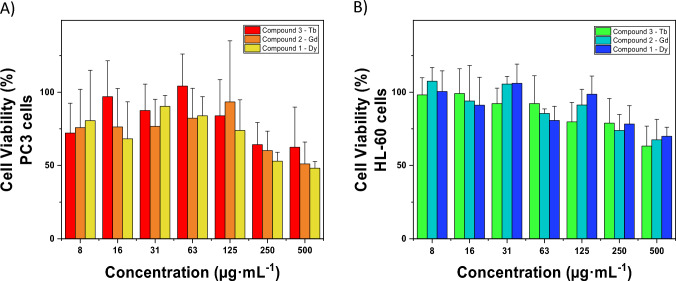
Cell viability of A) PC-3 cells and B) HL-60 after 24 h of incubation with **1–3**. Note that the shown data corresponds to the average of triplicate for each concentration, obtained in three independent experiments (a total of *n* = 9). Vertical error bars indicate calculated standard deviations

One of the current drawbacks of the antitumoral treatments is the associated side effects, thus alternative strategies to reduce toxicological concerns have been a challenging research aim [43, 44]. Taking this into account, together with the recognized antitumoral activity of both picolinate [20] and lanthanide ions/complexes (Dy, Gd, and Tb) [45–47], the safety and the antitumoral activity of the three obtained complexes were evaluated. First, their biocompatibility was assessed in human immunological cells HL-60, involved in relevant biological processes such as the cellular redox homeostasis or the activation of the immune system (e.g., reactive oxygen species, ROS, complement activation) [48, 49]. Lastly, their antitumor effect was investigated in a carcinogenic model, the prostatic PC-3 adenocarcinoma, selected as the most diagnosed male cancer and the 2nd highest cause of male cancer-related death [50]. After 24 h of exposure, both cell lines were influenced by the presence of these complexes regardless the metallic nature, observing a slightly higher toxicological effect with the higher complex doses in contact with the prostatic cells. In particular, cell viability decreased up to 50 ± 10% in the case of compounds **1** and **2**, and 60 ± 25% for **3** at the highest concentration (500 µg·mL^−1^ of all compounds; Fig. 5). Regarding their immunological impact, this metallic tendency was also noted but providing greatest biocompatible profile since the cellular viability was maintained ~ 70 ± 12%. IC50 calculated values are: Compound **1** = 427 ± 16 and 819 ± 12 µg·mL^−1^, Compound **2** = 538 ± 23 and 682 ± 11 µg·mL^−1^, Compound **3** = 654 ± 22 and 667 ± 15 µg·mL^−1^, for PC-3 and HL-60, respectively). Hence, one could suggest that these lanthanides-based complexes could provide potential effective antitumoral activity against tumoral cells in comparison with the non-tumoral nature.

In view of these circumstances, the cytotoxicity repercussion of each precursor was also assessed to shed light on the antitumoral mechanism of these materials. Thus, both the 5-npic linker along with the three lanthanide salts sources (Dy(NO_3_)_3_·6H_2_O, Gd(NO_3_)_3_·6H_2_O, and TbCl_3_·6H_2_O) were put in contact under the immunological and tumoral cellular scenario at the same proportion of each coordination complex. A high biocompatibility profile was obtained regardless the metallic precursors or chosen concentration, observing a slight reduction coming from the terbium, gadolinium and dysprosium hydrated salts (80 ± 20, 90 ± 13, and 100 ± 15% for TbCl_3_, Gd(NO_3_)_3_, and Dy(NO_3_)_3_, respectively; Fig. S6). In contrast, it should be noted that the strongest toxicity effect produced by 5-npic linker with a decrease of the tumoral cell viability up to ~ 40–50% in comparison with the non-tumoral (~ 60%). Thus, the main activity seems to be provided by the presence of the linker since 55 ± 9%, 40 ± 6% and 61 ± 6% of 5-npic for **1**, **2** and **3** were released in 30 min under PBS (reaching 100% of leakage after 5 h; see Fig. S4). Despite the free linker was also able to impact on the immunological cells, this effect seemed to be potentially decreased by its complexation with the lanthanide ions under the selected in vitro conditions (e.g., structural maintenance, delayed linker release). For those reasons, a higher antitumoral effect and lower cytotoxicity in immunological cells were observed for lanthanide-complexes compared to free ligand. Finally, further investigations are required to study the potential action mechanism depending on the complex composition and/or material stability (chemical and structural).

## Conclusions

Two novel lanthanide-complexes have been newly prepared and characterized based on Gd and Tb and 5-nitropicolinate, which are isostructural to a previous one published by us based on Dy: [Ln_2_(5-npic)_6_(H_2_O)_4_]·(H_2_O)_2_, where Ln = Dy **(1)**, Gd **(2)**, and Tb **(3)**.

Photoluminescent measurements revealed that the complexes exhibit ligand-centered emission probably due to π–π* electronic transitions in the aromatic ring of the ligand. On the other hand, all complexes showed anti-proliferative activity against the prostatic PC-3 adenocarcinoma cell line, regardless of the metallic nature with, however, a lower cytotoxic impact against healthy immunological cells. Considering that these are the first metal complexes based on Ln-ions with antiprostatic and luminescent properties, these results open the door of the development of novel Ln-complexes with potential combined treatment and diagnosis capabilities. Work can be anticipated in extending these intriguing results to novel 5-nitropicolinic acid with higher stability.

### Supplementary Information

Below is the link to the electronic supplementary material.

Supplementary file1 (PDF 417 KB)

## Data Availability

The data that support the findings of this study are available on request from the corresponding author.
