# Supplementary material for: Potential antiprostatic performance of novel lanthanide-complexes based on 5-nitropicolinic acid
Source: J Biol Inorg Chem. 2024 May 8;29(3):331–8. doi: 10.1007/s00775-024-02054-0 (PMC11111526; doi:10.1007/s00775-024-02054-0)
Supplement: Supplementary file 1 — Supplementary file1 (PDF 417 KB) [file 775_2024_2054_MOESM1_ESM.pdf]

Supplementary Information

Potential antiprostatic performance of novel lanthanide-complexes based on 5-nitropicolinic acid

Amalia García-García,[1 ‡] Pablo Cristobal-Cueto,[2 ‡] Tania Hidalgo,[2] Iñigo J. Vitórica-Yrezábal,[1] Antonio Rodríguez-Diéguez,[1] Patricia Horcajada,[2,*] Sara Rojas[1,*]

[1] Department of Inorganic Chemistry, Faculty of Science, University of Granada, Av. Fuente Nueva s/n, 18071 Granada, Spain

[2] Advanced Porous Material Unit, IMDEA Energy Institute, Av. Ramón de la Sagra 3, 28935 Móstoles, Madrid, Spain

‡ These authors contributed equally to this work

Table of content

S1. Crystallographic data of compounds 2 and 3 .... s2

S2. X-ray powder diffraction (XRPD).. s5

S3. Thermogravimetric analysis (TGA) s6

S4. Stability assays of compounds 1-3 .... s7

S5. *In vitro* biocompatibility of free ligand and lanthanide salts s8

S1. Crystallographic data of compounds 2 and 3

Table S1. Crystallographic data and structure refinement parameters of compounds **2** and **3**.

**Compound**	**2**	**3**
**Formula**	$C_{18}H_{15}GdN_6O_{15}$	$C_{18}H_{15}TbN_6O_{15}$
**M_r (g·mol^{-1})**	712.61	714.28
**CCDC**	2300374	2300375
**Crystal system**	Monoclinic	Monoclinic
**Space group**	$P2_1/c$	$P2_1/c$
**T (K)**	296.15	296.15
**a (Å)**	9.245(3)	9.2479(2)
**b (Å)**	29.727(10)	29.7262(7)
**c (Å)**	8.584(2)	8.5477(2)
**α (º)**	90	90
**β (º)**	90.448(10)	90.4770(10)
**γ (º)**	90	90
**V (Å^3)**	2359.1(13)	2349.72(9)
**Z**	4	4
**ρ_{calc} (g·cm^{-3})**	2.006	2.019
**μ (mm^{-1})**	2.903	3.102
**F(000)**	1396.0	1400.0
**Crystal size (mm^3)**	0.33x0.28x0.24	0.4x0.4x0.35
**Reflections collected**	36002	33617
**Independent reflections**	4855 [R_{int} = 0.0669, R_{sigma} = 0.0354]	4812 [R_{int} = 0.0450, R_{sigma} = 0.0252]
**GoF on F^2**	1.256	1.462
**Final R indexes [I > 2σ (I)]**	R_1 = 0.0423, wR_2=0.0878	R_1 = 0.0328, wR_2=0.0732
**Final R indexes [all data]**	R_1 = 0.0492, wR_2=0.0945	R_1 = 0.0344, wR_2=0.0738

Table S2. Bond lengths (Å) and angles (°) of coordination spheres for **2**.

Bond	Distance (Å)	Atoms	Angle (°)
Gd1-O1	2.489(4)	O1-Gd1-O1$^{(i)}$	66.35(15)
Gd1-O1$^{(i)}$	2.407(4)	O1-Gd1-N1	64.15(14)
Gd1-O4	2.296(4)	O1$^{(i)}$-Gd1-N1	129.66(14)
Gd1-O8	2.341(4)	O1-Gd1-N4	139.74(15)
Gd1-O10	2.345(4)	O1$^{(i)}$-Gd1-N4	112.77(14)
Gd1-O11	2.366(4)	O4-Gd1-O1$^{(i)}$	134.01(14)
Gd1-N1	2.558(5)	O4-Gd1-O1	139.41(14)
Gd1-N4	2.655(5)	O4-Gd1-O8	137.60(15)
		O4-Gd1-O10	81.27(15)
		O4-Gd1-O11	76.21(15)
		O4-Gd1-N1	83.12(15)
		O4-Gd1-N4	74.83(15)
		O8-Gd1-O1$^{(i)}$	79.69(14)
		O8-Gd1-O1	70.35(14)
		O8-Gd1-O10	79.87(16)
		O8-Gd1-O11	145.13(14)
		O8-Gd1-N1	92.02(16)
		O8-Gd1-N4	63.89(14)
		O10-Gd1-O1$^{(i)}$	81.51(14)
		O10-Gd1-O1	139.09(14)
		O10-Gd1-O11	121.73(17)
		O10-Gd1-N1	146.13(16)
		O10-Gd1-N4	75.96(16)
		O11-Gd1-O1$^{(i)}$	77.41(15)
		O11-Gd1-O1	76.49(14)
		O11-Gd1-N1	82.87(16)
		O11-Gd1-N4	142.83(15)
		N1-Gd1-N4	71.00(15)
		Gd1-O1-Gd1$^{(i)}$	113.65(15)

*Symmetry operation: (i) 1-x, 1-y, -z

Table S3. Bond lengths (Å) and angles (°) of coordination spheres for **3**.

Bond	Distance (Å)	Atoms	Angle (°)
Tb1-O1	2.347(3)	O1-Tb1-O2$^{(i)}$	77.51(11)
Tb1-O2$^{(i)}$	2.405(3)	O1-Tb1-O2	76.56(11)
Tb1-O2	2.471(3)	O1-Tb1-N1	82.85(12)
Tb1-O7	2.285(3)	O1-Tb1-N2	142.75(11)
Tb1-O10	2.326(3)	O2-Tb1-O2$^{(i)}$	66.40(12)
Tb1-O13	2.330(3)	O2-Tb1-N1	64.50(10)
Tb1-N1	2.545(4)	O2$^{(i)}$-Tb1-N1	130.03(11)
Tb1-N2	2.648(4)	O2-Tb1-N2	112.91(11)
		O2$^{(i)}$-Tb1-N2	139.73(11)
		O7-Tb1-O1	76.12(11)
		O7-Tb1-O2$^{(i)}$	133.94(11)
		O7-Tb1-O2	139.45(11)
		O7-Tb1-O10	81.22(11)
		O7-Tb1-N1	82.82(12)
		O7-Tb1-N2	74.76(12)
		O7-Tb1-O13	137.59(11)
		O10-Tb1-O1	121.44(13)
		O10-Tb1-O2$^{(i)}$	81.28(11)
		O10-Tb1-O2	139.07(10)
		O10-Tb1-N1	147.15(12)
		O10-Tb1-N2	76.12(12)
		O10-Tb1-O13	79.70(12)
		N1-Tb1-N2	70.95(12)
		O13-Tb1-O1	145.35(11)
		O13-Tb1-O2	70.58(11)
		O13-Tb1-O2$^{(i)}$	79.51(11)
		O13-Tb1-N1	92.60(12)
		O13-Tb1-N2	64.04(11)
		Tb1-O2-Tb1$^{(i)}$	113.60(12)

*Symmetry operation: (i) 1-x, 1-y, 2-z

S2. X-ray powder diffraction (XRPD)

XRPD technique was carried out to investigate the reproducibility and purity of samples. XRPD patterns were collected at room temperature.

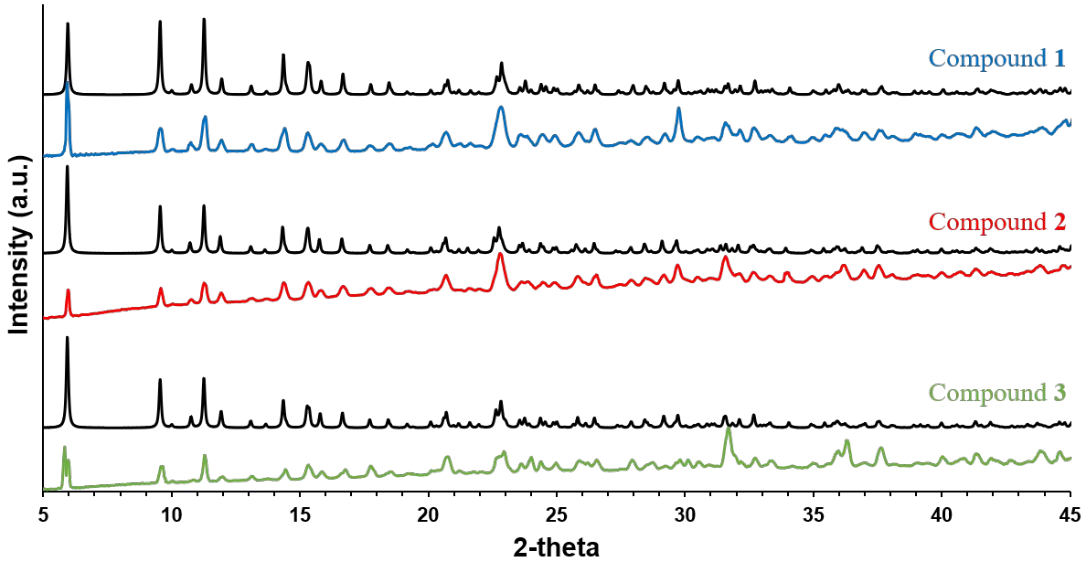

Fig. S1. Experimental XRPD patterns of compounds **1-3** (in blue, red, and green color) and their corresponding simulated patterns (in black).

S3. Thermogravimetric analysis (TGA)

The TGA spectra of compounds **1-3** are slightly similar. Along the two first steps from room temperature to 240 ºC the weight loss are attributed to the two crystallization and four coordinated water molecules. The following mass loss starting at 300 ºC could correspond to the lanthanide complexes decomposition.

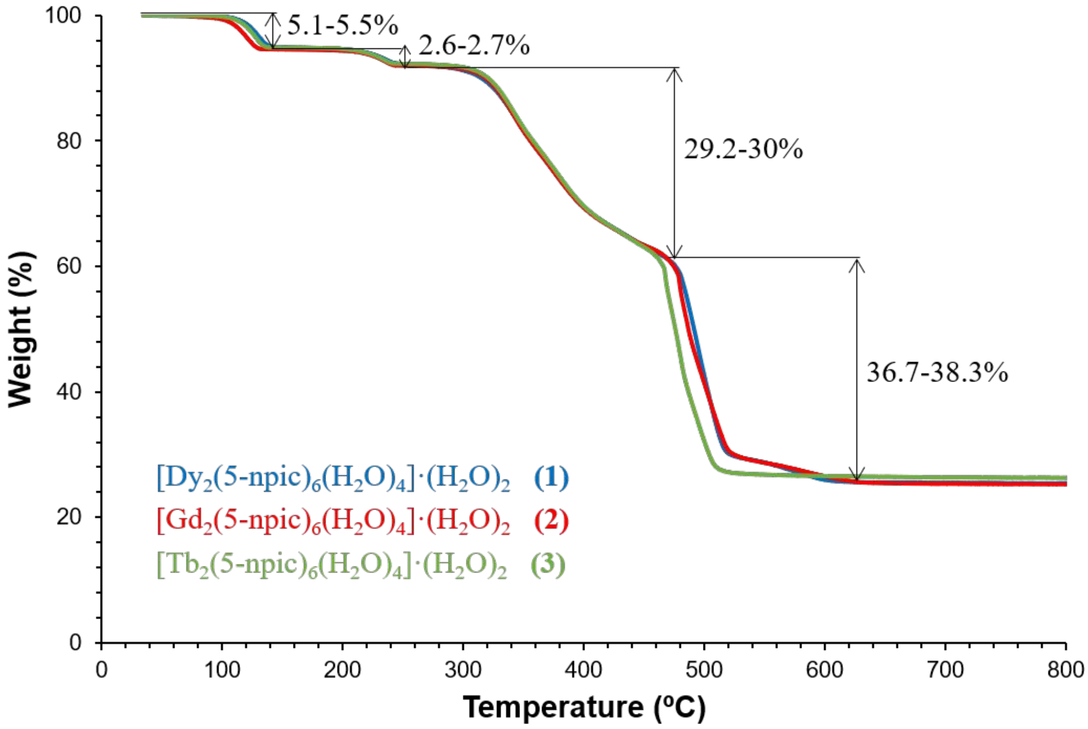

Fig. S2. TGA curves of compounds **1-3** at a heating rate of 10 ºC·min^{-1} and an airflow of 50 mL·min^{-1}.

S4. Stability assays of compounds 1-3

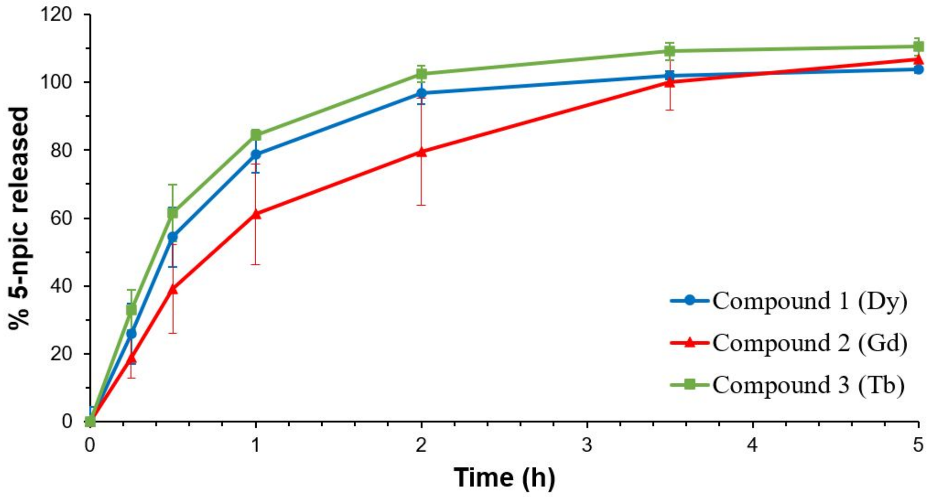

Fig. S3. Degradation profile of complexes **1-3** in PBS at 37 ºC under sink conditions. Error bars show standard deviations from triplicate experiments (n = 3).

The degradation data of compounds **1-3** were attempt to fit to zero-, first- and second-order kinetic, but they are only well described by zero-order kinetics according to Eq. 1:

$$\textbf{Eq. 1}: \ [\%C] = Kt + [\%C]_0$$

where [%C] and $[\%C]_0$ are the percentage of total amount of released 5-npic ligand at the time t (h) and the initial % 5-npic, respectively (when t = 0, $[\%C]_0 = 0$), and K is the zero-order kinetics constant.

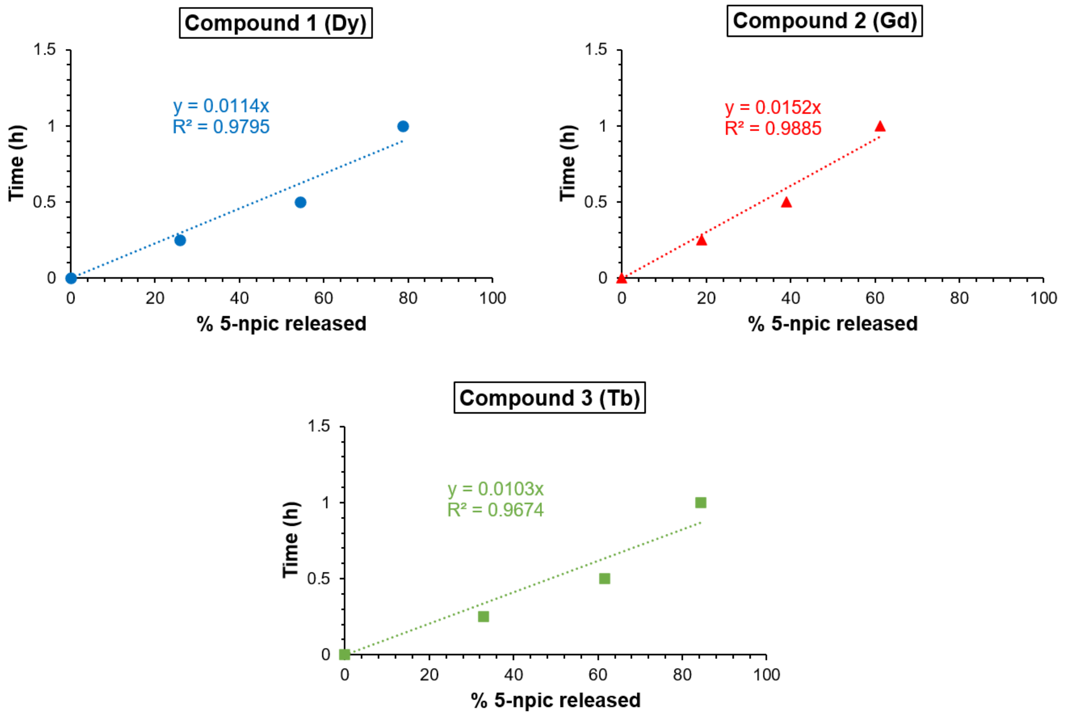

Fig. S4. Fitting analysis of released 5-npic (%) in complexes **1-3** to a zero-order kinetics.

S5. *In vitro* biocompatibility of free ligand and lanthanide salts

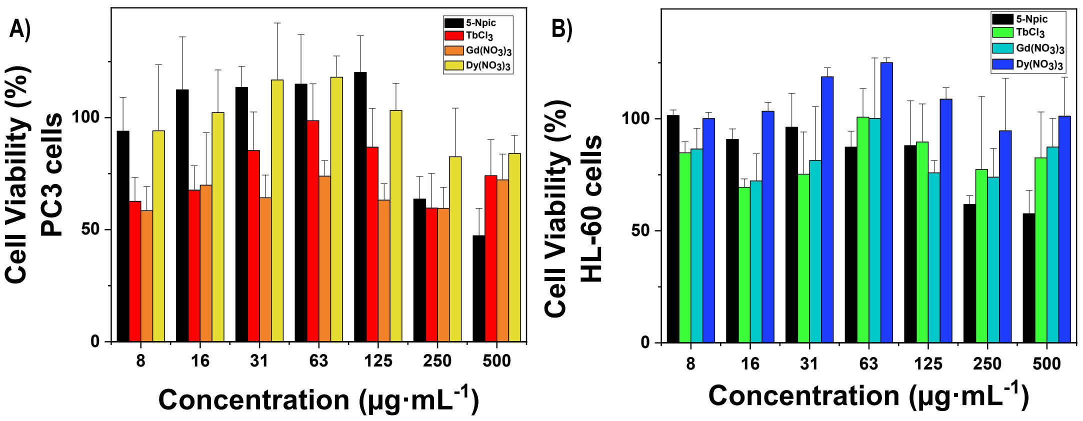

Fig. S5. Cell viability of A) PC-3 cells and B) HL-60 after 24 h of incubation with a concentration range of each lanthanide's precursors: 5-npic linker and the three metallic sources ($Dy(NO_3)_3$, $Gd(NO_3)_3$, and $TbCl_3$). Note that the shown data corresponds to the average of triplicate for each concentration, obtained in two independent experiments (total nº = 9). Vertical error bars indicate calculated standard deviations.
